# Contributing roles of mitochondrial dysfunction and hepatocyte apoptosis in liver diseases through oxidative stress, post-translational modifications, inflammation, and intestinal barrier dysfunction

**DOI:** 10.1007/s00018-023-05061-7

**Published:** 2024-01-12

**Authors:** Karli R. LeFort, Wiramon Rungratanawanich, Byoung-Joon Song

**Affiliations:** Section of Molecular Pharmacology and Toxicology, National Institute on Alcohol Abuse and Alcoholism, 9000 Rockville Pike, Bethesda, MD 20892 USA

**Keywords:** Alcohol-associated liver disease, Metabolic dysfunction-associated steatotic liver disease, Drug-induced liver injury, Viral hepatitis, Oxidative stress, CYP2E1, ALDH2, Post-translational modifications, Mitochondrial dysfunction, Apoptosis, Intestinal barrier dysfunction, Microbiota, Endotoxemia, Gut–liver axis

## Abstract

This review provides an update on recent findings from basic, translational, and clinical studies on the molecular mechanisms of mitochondrial dysfunction and apoptosis of hepatocytes in multiple liver diseases, including but not limited to alcohol-associated liver disease (ALD), metabolic dysfunction-associated steatotic liver disease (MASLD), and drug-induced liver injury (DILI). While the ethanol-inducible cytochrome P450-2E1 (CYP2E1) is mainly responsible for oxidizing binge alcohol via the microsomal ethanol oxidizing system, it is also responsible for metabolizing many xenobiotics, including pollutants, chemicals, drugs, and specific diets abundant in n-6 fatty acids, into toxic metabolites in many organs, including the liver, causing pathological insults through organelles such as mitochondria and endoplasmic reticula. Oxidative imbalances (oxidative stress) in mitochondria promote the covalent modifications of lipids, proteins, and nucleic acids through enzymatic and non-enzymatic mechanisms. Excessive changes stimulate various post-translational modifications (PTMs) of mitochondrial proteins, transcription factors, and histones. Increased PTMs of mitochondrial proteins inactivate many enzymes involved in the reduction of oxidative species, fatty acid metabolism, and mitophagy pathways, leading to mitochondrial dysfunction, energy depletion, and apoptosis. Unique from other organelles, mitochondria control many signaling cascades involved in bioenergetics (fat metabolism), inflammation, and apoptosis/necrosis of hepatocytes. When mitochondrial homeostasis is shifted, these pathways become altered or shut down, likely contributing to the death of hepatocytes with activation of inflammation and hepatic stellate cells, causing liver fibrosis and cirrhosis. This review will encapsulate how mitochondrial dysfunction contributes to hepatocyte apoptosis in several types of liver diseases in order to provide recommendations for targeted therapeutics.

## Introduction

Liver diseases are named based on their etiology. The hepatotoxicity of some drugs causes drug-induced liver diseases (DILI) and alcohol-associated liver disease (ALD) is a harmful consequence that may occur from excessive and chronic alcohol consumption. Liver diseases can also occur from overnutrition and obesity [metabolic dysfunction-associated steatotic liver disease (MASLD)], toxic agents [toxicant-associated fatty liver disease (TAFLD)], and hepatitis viruses (viral hepatitis). However, liver diseases, regardless of the cause, share many pathophysiological features like oxidative stress, post-translational modifications (PTMs), fat accumulation, metabolic signaling alterations, mitochondrial dysfunction, gut barrier dysfunction, inflammation, and hepatocyte apoptosis. Overall, we have reviewed the cellular and molecular pathologies of these liver diseases to derive potential preventions or therapeutic targets against liver diseases [1].

### Oxidative stress in liver diseases

Oxidative stress is an imbalance between pro-oxidants and antioxidants. Usually, the cell can remove reactive oxygen species (ROS) and reactive nitrogen species (RNS) through antioxidant molecules such as reduced glutathione (GSH) and primary defense enzymes such as superoxide dismutase (SOD), catalase (CAT), and glutathione-dependent peroxidases (GPx) [1]. So why has oxidative stress been observed in studies on DILI [2–5], TAFLD [6–8], ALD [9–12], and MASLD [13–23]? When organelles such as mitochondria, endoplasmic reticula (ER), and peroxisomes are damaged and dysfunctional, more ROS are produced [1, 18] in a vicious cycle [14, 24]. Excessive oxidative stress may cause DNA oxidation [25], lipid peroxidation, oxidative protein modifications [26], impaired fat metabolism [6, 20, 26–28], systemic inflammation [26, 29, 30], and tissue damage [31–33], all of which contribute to the progression of liver diseases [17, 34, 35].

Hepatocytes respond to oxidative stress in diverse ways. Hepatic stellate cells (HSCs), when activated by ROS and damage-associated molecular patterns (DAMPs) from injured or necrotic hepatocytes, will produce extracellular matrix components to construct fibrotic tissue [36]. On the other hand, Kupffer cells, upon activation by endotoxins (including lipopolysaccharide, LPS) or superoxide anions (⋅O_2_^−^), will then produce more ROS through stimulation of NADPH-dependent oxidases (NOXs), and the redox-sensitive transcription factor nuclear factor-κB (NF-κB)-mediated pro-inflammatory storm of cytokines, chemokines, and cell adhesion molecules (CAMs) [36]. Consequently, in response to oxidative stress, hepatocytes stimulate necrotic and apoptotic pathways, leading to impaired liver function, worsened paracrine inflammation, and fibrogenesis [36].

### Post-translational protein modifications in liver diseases

PTMs regulate the localization, stability, and final activity of virtually all proteins in the context of liver diseases [37, 38]. The proteins involved in promoting various PTMs exist in several subcellular organelles, including the cytoplasm [39], ER [40, 41], mitochondria [42], and nucleus [43]; PTMs may also be observed in the proteomes of the liver [37, 44–47], gut [7], and other peripheral tissues [29]. PTMs found in liver diseases include protein acetylation [37, 42, 48], nitration [10, 26, 37, 49–53], *S*-nitrosylation [49, 54, 55], oxidation [37], phosphorylation [26, 56, 57], succinylation [58], ADP-ribosylation [44], ubiquitination [37, 59], SUMOylation [60–65], carbonylation [66–68], *S*-palmitoylation [45–47, 69], glycosylation [7, 37, 70, 71], protein adducts of aldehyde (i.e., acetaldehydes) [9, 72], lipid peroxidation products (LPOs), and advanced glycation end products (AGEs) [6, 66, 74–79]. Several studies detail that specific PTMs correlate with exposures to excessive alcohol [80, 81], CCl_4_ [7, 56], acetaminophen (APAP) [53, 82], 3,4-methylenedioxymethamphetamine (MDMA, Ecstasy) [39], or fructose [51]. In these cases, PTMs accumulate in cells and contribute significantly to fat accumulation [54], hepatocyte apoptosis, inflammation, fibrosis [51], and cirrhosis [37], by altering signaling pathways affiliated with liver disease progression [43, 66, 67] and by targeting proteins for ubiquitin-mediated proteolysis [59]. Overall, PTMs are one of the various ROS-mediated changes that occur on cellular and translational levels [29] to, directly and indirectly, contribute to alcohol-mediated hepatic injuries [12, 83, 84]. The contributing roles of specific PTMs or simple consequences in the disease process can be elucidated by carefully studying their time-dependent events. The functional activities of a specific PTM on a few selected proteins should also be found in disease models to figure out their roles further. Based on the concept and approaches, precisely characterizing the roles of specifically targeted PTMs in designated subcellular organelles or tissues can provide valuable information to understand better the molecular mechanisms of liver diseases or even genetic- and aging-related disorders. For example, in various alcohol exposure models, an elevated intestinal activity or expression of the ethanol-inducible cytochrome P450-2E1 (CYP2E1) contributes to increased ROS/RNS [12, 85], promoting PTMs (e.g., nitration [26, 51, 52, 59, 68, 86]), which structurally alters the intestinal environment creating inflammation, gut tight junction (TJ) and adherens junction (AJ) protein degradation, apoptosis of enterocytes in the intestines [11, 86, 87], systemic endotoxemia, and the progression of ALD  [88, 89].

### Mitochondrial dysfunction in liver diseases

Mitochondria are critical sites of bioenergetics, fat oxidation, intermediary metabolism, apoptosis, mitophagy, and redox homeostasis [90–93]. They are one of the primary sources of oxidative stress involved in fatty liver diseases [94]; α-ketoglutarate dehydrogenase and pyruvate dehydrogenase are involved in redox reactions to generate NADH and FADH_2_ in the tricarboxylic acid (TCA) cycle [95], then in the electron transport chain (ETC), complexes I, II, and III perform redox reactions to generate ATP [18]. Thus, to balance out the ROS/RNS generated from these redox reactions, many mitochondrial antioxidants and enzymes exist.

Mitochondrial antioxidant proteins in the first line of defense against ROS/RNS fall into three categories, including non-enzymatic antioxidants (i.e., GSH), direct enzymatic antioxidants (i.e., SOD2 and GPx), and indirect enzymatic antioxidants [i.e., glutathione reductases (GR), peroxiredoxins, and thioredoxins (Trx)] [96, 97]. The transcription of many of these antioxidants is either regulated by peroxisome proliferator-activated receptor-γ coactivator-1α (PGC-1α) [98] or by nuclear factor erythroid 2-related factor (Nrf2) separating from its regulatory binding protein Kelch-like ECH-associated protein (Keap1) [99, 100]. Typically, in the second line of defense, mitochondria trigger mitophagy to restore redox homeostasis and retain the function of other mitochondria and organelles for cellular activities [101]. However, these natural lines of defense in mitochondria can be altered after excessive consumption of or exposure to alcohol (ethanol) [85, 94], drugs [66, 102, 103], viruses [104], and some nutrients, including fructose [105]. Specifically, these exposures can alter mitochondrial redox homeostasis and oxidatively damage DNA [106, 107], RNA [106], lipids, and proteins, through PTMs [8, 26, 48, 70, 108, 109], causing inactivation of their functions and signaling pathways, leading to mitochondrial dysfunction [26, 53, 68, 83], death of hepatocytes, and liver damage [17, 110] or age-related conditions [91, 111–113]. Not only can many types of PTMs accumulate in mitochondria [85], but they can also downregulate the expression of or inactivate mitochondrial deacetylases like sirtuin 3 (SIRT3) [114–117], sirtuin 4 (SIRT4) [118–121], and sirtuin 5 (SIRT5) [8, 48,108, 109, 117, 122, 123] in the context of ALD [124, 125], MASLD [51, 126, 127], and other conditions [128]. Nucleus-localized sirtuin 1 (SIRT1) migrates to mitochondria to exert its effects [125] and is another prevalent target in the context of ALD [125, 129–133] and MASLD [22, 117, 133]. Other than sirtuin pathways, signaling pathways of PGC-1α [131, 134], AMPK [135, 136], as well as Bax and Bcl2 [132, 137], are altered to cause mitochondrial dysfunction and apoptosis of hepatocytes in liver diseases [131, 132, 134–137]. However, these proteins represent important targets for prevention and therapy [75, 85, 138–140]. Overall, mitochondrial conditions likely provide a more comprehensive picture of the molecular pathology of liver diseases [75, 127, 141, 142].

### Intestinal barrier dysfunction in liver diseases

The gut, like many organs, has multiple barriers; the immunological barrier is composed of gut-associated lymphoid tissue, the physical barrier consists of epithelial TJ/AJ proteins and microbiota, and the chemical barrier is composed of antimicrobial proteins, IgA, and a mucus layer [143]. Gut dysbiosis is a term to describe changes in the relative abundance of beneficial and pathogenic bacteria, where excessive Gram-negative bacteria may stick to and cause perforations in the gut barrier wall (intestinal permeability changes) and “leak” various toxic metabolites [e.g., bile acids, trimethylamine (TMA), and LPS, a cell wall component of Gram-negative bacteria] into systemic circulation [144]. The atypical transmission of pathogenic gut bacteria and toxic metabolites (endotoxemia) acts to permeate the barriers of the liver through the portal vein, enterohepatic circulation, or bile acid secretions [145–147]. Gut dysbiosis is inextricably linked to the exacerbation and progression of various liver diseases [148–153]. Altered microbiota compositions have been found in several clinical cases of ALD [154, 155], cirrhosis [156–159], MASLD [160, 161], viral hepatitis including Hepatitis B viral infections (HBV) [162–165] and Hepatitis C viral infections (HCV) [166–168], as well as hepatocellular carcinoma (HCC) [158, 169]. Furthermore, mechanistic animal studies on hepatic endotoxemia have shown it to be positively correlated with intestinal barrier dysfunction; elevated PTMs that disrupt TJ/AJ protein networks allow intestinal contents such as pathogenic bacteria and LPS to leak out into systemic circulation, characteristically manifesting in hepatic nitro-oxidative stress [170–172]. Endotoxemia is also affiliated with mitochondrial dysfunction [25] and systemic inflammation [172] in rodent models of ALD [11, 126, 151, 173] and MASLD [20, 51, 68, 151, 174–178]. Sometimes, the etiology of ALD and MASLD may even overlap; several reports detail several taxa that produce endogenous ethanol, as observed in experimental models [51, 179], adult patients with MASLD [160], and even young children with metabolic dysfunction-associated steatohepatitis (MASH) [180]. Some cases of ALD [126] and MASLD [181–183] may even affect other peripheral organs via the intestinal route.

## The causal roles of oxidative stress, PTMs, mitochondrial dysfunction, and intestinal barrier dysfunction in promoting several chronic and acute liver diseases

### Alcohol-associated liver disease

#### Alcohol-mediated oxidative stress and gut barrier dysfunction

Alcohol is first absorbed in the small intestines, then transported to the liver and metabolized mainly by oxidative and non-oxidative metabolism [184–186]. The majority of alcohol is oxidized in the cytosol by aldehyde dehydrogenase (ADH), in peroxisomes by CAT, and in microsomes and mitochondria by CYP2E1 via the microsomal ethanol-oxidizing system (MEOS) before toxic acetaldehyde is further detoxified by mitochondrial aldehyde dehydrogenase 2 (ALDH2) [187, 188]. Liver diseases have been widely studied in the context of these enzymes that either oxidatively metabolize ethanol into toxic metabolites, or detoxify alcohol and are made less effective through genetic polymorphisms. It is known that oxidative damage from NAPDH-dependent CYP2E1 metabolism [189] leads to toxicological damage in ALD [190], causing events such as increased ROS production [190], antioxidant downregulation, mitochondrial dysfunction [191, 192], suppressed fatty acid oxidation, oxidative DNA damage, and protein adduct formation via lipid peroxidation [i.e., acrolein, malondialdehyde (MDA), and 4-hydroxynonenal (4-HNE)] in the liver [193]. In addition, another study showed a crucial role of CYP2E1 in alcohol-mediated oxidative DNA damage in the liver [194].

However, there is evidence that alcohol absorption along with oxidative and non-oxidative metabolism, occurs in the gut [187, 195]. Studies have found the expression of ADH 4 isoform [196], CYP2E1 [87], and ALDH2 in the intestines [87], as well as inactivated ALDH2 [55, 83] and upregulated CYP2E1 [87], acetaldehyde, and LPS [55, 83, 87, 196] in models of ALD. These changes indicate signs of oxidative ethanol metabolism that results in alcohol-induced oxidative stress and intestinal barrier dysfunction. Recently, it has been shown that protective ALDH2 is inactivated through PTMs such as oxidation [55]  and *S*-nitrosylation in ALD [55, 83], nitration in APAP-mediated DILI [53, 59], phosphorylation in CCl_4_-mediated TAFLD [56, 197], oxidation in MDMA-induced TAFLD [198], and lipid peroxidation products in ALD [199]. Some believe that acetaldehyde independently causes alcohol-associated organ damage; one study displayed that it independently disrupted intestinal TJ and AJ protein networks, leading to endotoxemia and liver injury [9]. Similar effects of acetaldehyde have been shown in models of chronic alcohol exposure in *Aldh2*-KO mice, resulting in ALD [200]. Other studies conducted with binge alcohol-exposed *Aldh2*-KO mice suggest that acetaldehyde stimulates intestinal barrier dysfunction, leading to acute liver injury [10].

When ALDH2 activity is depleted in *Aldh2*-KO mice [201], pro-oxidant CYP2E1 has been found to be upregulated in the intestines upon alcohol exposure [87, 201, 202]. Intestinal CYP2E1-mediated oxidative stress can also sensitize the liver to toxicity through endotoxemia and gut-derived TNF-α through the CYP2E1-thioredoxin-ASK1-JNK1 pathway [203]. The effects of intestinal CYP2E1 on gut barrier dysfunction and endotoxemia are worsened with concomitant LPS administration [11]. Hepatic CYP2E1 is also another significant contributor to alcohol-induced oxidative stress and signaling pathway alterations [9], as shown in multiple studies examining the effects of inhibiting or knocking out the *Cyp2e1* gene for [204–206]. For example, polyenyl phosphatidylcholine (PPC) effectively suppressed alcohol-mediated oxidative stress and then was found to be inhibiting CYP2E1 [40, 207]. In another study, transgenic over-expression of CYP2E1 in mice exacerbated the pathogenesis of ALD [206, 208] and MASLD [209, 210]. Perhaps reversing the expression and activities of CYP2E1 and ALDH2 may serve a purpose in protecting against alcohol-induced hepato-intestinal oxidative stress and gut barrier dysfunction. For example, a translational study showed that ALDH2 suppression was protected by physiologically relevant levels of omega-3 polyunsaturated fatty acids [103, 211], and in a recent phase II clinical trial, treatment with clomethiazole (CMZ), an inhibitor of CYP2E1, mitigated the biomarkers of alcohol-induced oxidative stress and ALD progression [212].

#### Alcohol-mediated oxidative stress and mitochondrial dysfunction in the fatty liver

Alcohol-mediated steatosis (or fat accumulation) can be induced by activating de novo fat synthesis, blocking fat degradation pathways, or increasing the transport of lipids from other tissues [190]. On a molecular level, this may also occur through mitochondrial dysfunction associated with decreased fat degradation due to PTM-mediated suppression of the enzymes in β-oxidation [103, 211], upregulated immune cell infiltrations, protein adducts, lipid peroxidatifon, and DNA damage [190].Alcohol-mediated ROS and RNS can also downregulate β-oxidation activity by inhibiting peroxisome proliferator-activated receptor-α (PPAR-α) and a lipid catabolism regulator named AMP-activated protein kinase (AMPK) [9, 213] while upregulating sterol regulatory binding protein-1 (SREBP-1) to increase hepatic fatty acid and cholesterol biosynthesis [9, 213]. Furthermore, chronic and binge alcohol models show increased lipid transport to the liver, contributing to fat accumulation in hepatocytes [214, 215]. A study from Ceni et al. theorizes that in cases of ALD, steatosis may happen epigenetically by targeting forkhead box (FoxO3a) and SIRT1, which serve as intermediaries between autophagy and transcriptional lipid metabolism regulation [9].

#### The vicious cycle of oxidative stress and inflammation in promoting fibrosis

Oxidative stress and inflammation reciprocally communicate in a positive feedback loop to stimulate fibrosis. The accumulation of ROS and LPOs promotes hepatocyte apoptosis/necrosis, which activates hepatic stellate cells (HSCs) [190]. In an attempt to heal this “wound,” HSCs may promote fibrosis and cirrhosis in an inflammatory signaling response [190]. The ROS-mediated activation of HSCs can be seen through the accumulation of α-smooth muscle actin (α-SMA), vimentin (VIM), and collagen in the extracellular matrices of hepatocytes [9, 191, 192]. Fibrosis may also be stimulated by upregulating the MDA/4-HNE pathway; this pathway will, in turn, upregulate pro-fibrogenic matrix metalloproteinase-2 (MMP2) and downregulate matrix metalloproteinase-1 (MMP1), two enzymes responsible for remodeling the hepatic extracellular matrix [9, 191, 192, 216]. Another method of alcohol-induced ROS-mediated liver fibrosis happens when acetaldehyde and other reactive aldehydes upregulate the expression of fibrogenic transforming-growth-factor-β (TGF-β). This stimulates the production of collagen and α-SMA and decreases interferon-γ signaling in HSCs to cause fibrosis [9]. Other PTMs, such as acetylation and methylation, have been known to contribute to the progression of fibrosis in ALD [72, 195, 217, 218]. This accumulated inflammation is worsened by increased intestinal barrier dysfunction [144, 219]. The likely-resulting endotoxemia from intestinal barrier dysfunction stimulates toll-like receptor 4 (TLR4) in Kupffer cells to produce NADPH-oxidase (NOX)-dependent ROS [213] and activate HSCs, leading to fibrosis [144, 219]. Additionally, the efforts of Nrf2 and its downstream antioxidant enzymes and proteins may be exhausted or suppressed, leading to lowered antioxidant levels, in the progression of liver injury to hepatitis and fibrosis [220].

### Metabolic dysfunction-associated steatotic liver disease

MASLD is closely affiliated with obesity [221, 222], type-2 diabetes mellitus (T2DM) [221, 222], hypertension [221], and metabolic syndrome [221]. MASLD may be further specified as TAFLD. However, it should be noted that the overall mechanism of MASLD shares many commonalities with that of ALD. In MASLD and ALD, dysregulated lipid metabolism contributes to lipotoxicity and peroxidation [223], leading to ER stress, mitochondrial dysfunction, and hepatocyte damage. These alterations activate HSCs, leading to inflammation and fibrogenesis [193, 223–225]. Liver insults begin with the phase of steatosis; mitochondrial respiration increases to meet the increased need for energy. As a result, ROS production increases, activating antioxidant responses [95, 226, 227]. Lipid accumulation will develop due to the excess of free fatty acids (FFAs) [223], compromising mitophagy responses through the activation of JNK-dependent apoptosis [95, 226, 227]. In the next stage of MASH, inflammation and oxidative stress occur in a vicious, positive feedback loop [228], triggering apoptosis of hepatocytes [227], and potentially compromising cellular respiration, mitophagy, and antioxidant pathways. Fibrosis is initiated when increased inflammation, oxidative stress, and hepatocyte apoptosis cumulatively stimulate Kupffer cells and HSCs, as well as neutrophil infiltration, to repair the “wounds” [95, 229].

Despite this common pathology of ALD and MASLD, the Multiple-Hit Hypothesis remains a phenomenon more studied in the context of MASH/MASLD. The Multiple-Hit Hypothesis details increased fat accumulation to be the “first hit” [230]. According to Day and James, oxidative stress follows steatosis as the “second hit” [230]. MASLD has many manifestations of metabolic syndrome, from as little as lipid droplets to total systemic inflammation seen in MASH, with the possibility of progression to fibrosis and HCC. Other factors, such as inflammation, altered hepatocyte apoptosis signaling, and activation of HSCs, allow milder cases of MASLD to progress to more severe cases, including MASH [14]. More reports mention the  Three-Hit Hypothesis, involving dysregulated lipid metabolism, mitochondrial dysfunction with decreased fat degradation, and oxidative stress that happens in cycles in the progression of MASLD cases [231, 232]. Additional theories on the Multiple-Hit Hypothesis of MASLD detail the role of lipid and sugar metabolism, gut barrier dysfunction, and systemic inflammation as common factors in metabolic syndrome [233]

MASLD/MASH, caused by non-alcohol substances, such as fructose/sucrose and Western-style high-fat diets (HFDs) (containing high ratios of pro-inflammatory omega-6 fatty acids to anti-inflammatory omega-3 fatty acids), is a hepatic manifestation of metabolic syndrome [234]. Similar to ALD, increased de novo fat synthesis and fat transport from adipose tissue with decreased mitochondrial fat degradation are usually observed in MASLD [235]. Increased oxidative stress and nitrative stress also significantly contribute to the progression of MASLD/MASH. Oxidative stress may happen partly through the involvement of translocation and activation of mitochondrial NOX4 [235], CYP2E1-generated ROS [68, 210, 236], HFD-mediated insulin resistance [22], and various PTMs of mitochondrial proteins, causing mitochondrial dysfunction, decreased fat degradation, and elevated hepatocyte death [49].

#### The effect of fat metabolism dysregulation on oxidative stress

Elevated FFAs can be more hepatotoxic than TG accumulation since FFAs can cause JNK-mediated hepatocyte apoptosis [227] and the production of a cytokine storm in the progression of MASLD and MASH [226]. In addition, an increased FFA pool may lead to oxidative stress in cells, altering apoptosis and causing NF-κB-related inflammatory pathways that induce cytokine production and activate HSCs [235]. The specific mechanism of FFA and ROS-related apoptosis of hepatocytes involves the regulation of the mitochondria by Bcl-2 and Bax. For instance, when NOX4 and CYP2E1 oxidize substrates like long-chain FFAs, uncoupled electrons leak from the mitochondrial ETC [68]. When FFAs are oxidized in peroxisomes and the ER, oxidative stress accumulates and activates the Bax/Bcl-2 complex through FoxOa3 and JNK [237]. Bax, when released from Bcl-2, will induce a mitochondrial permeability transition (MPT) in response to this oxidative stress [238], which will trigger cytochrome c to be released from mitochondria, activating caspase-mediated apoptosis [235]. Alternatively, activated JNK can stimulate the phosphorylation of Bax, leading to its translocation to mitochondria to cause mitochondrial permeability changes and hepatocyte apoptosis [239].

Besides the accumulation of FFAs and LPOs, cholesterol (dys)regulation significantly affects the onset and progression of MASLD. One study suggests that the cholesterol-to-bile acid ratio is vital to supporting the homeostatic redox environment of HSCs [240]. One report showed that cholesterol could be a selective inducer of oxidative stress and mitigate fibrosis by inducing HSC apoptosis [240]. A recent genetic study suggested that having a good cholesterol index (i.e., more high-density lipoprotein (HDL) cholesterol and less low-density lipoprotein (LDL) cholesterol) could significantly prevent FLD because HDLs allow LDLs and other fats to be filtered through the liver and excreted rather than accumulating in blood vessels and tissues [241].

#### The effect of insulinemia on fat metabolism and inflammation

Insulin resistance/insulinemia, genetics, and metabolic syndrome account for most cases of MASLD [231, 235]. Insulinemia has been shown to serve as a cause of FFA accumulation [242]. Mainly, most FFAs in hepatocytes are found in the FFA pool in the liver and transported after lipolysis from other tissues [242]. Insulin typically signals lipolysis of triglycerides (TGs) into FFAs. However, in cases of insulinemia, adipose cells are in constant lipolysis, causing excess FFAs to travel to the liver and lead to fat accumulation [18, 235].

Other studies have detailed an interaction between the NF-κB pathway and insulin resistance to MASLD [19]. Many therapeutic effects against MASLD related to oxidative stress and lipid peroxidation have been explored through this pathway, including normalizing mitochondrial function with proper mitochondrial β-oxidation and ATP synthesis [19]. Inhibiting the redox-sensitive transcription factor NF-κB is also an essential therapy targeting inflammation in MASLD to prevent the progression to worse disease stages such as MASH [19].

#### The effect of oxidative stress on fat metabolism and inflammation

Recent reviews suggest that increased oxidative stress may cause de novo lipogenesis through upregulation of SREBP-1 and mitochondrial dysfunction in MASLD [24, 228]. In MASH, ROS primarily come from mitochondrial electron leakage, pro-oxidative enzyme activation (i.e., CYP2E1, NOX4), iron accumulation and Fenton reaction metabolism [18, 243], and antioxidant depletion [235]. While it is well documented that CYP2E1 contributes to oxidative stress pathways in ALD and DILI, many reports have also demonstrated the critical role of CYP2E1 in MASLD through the production of ROS and LPOs; this may represent the second hit in the progression of steatosis to MASH [26, 41, 68, 178, 244]. Thus, this oxidative and lipotoxic stress must be balanced out with the help of various antioxidants.

The Nrf2/ARE pathway is a crucial prevention for MASLD because it counteracts oxidative stress and corrects lipid metabolism [19]. Under physiological states, Nrf2, a transcription factor, is usually bound to Keap1. Under oxidative stress conditions, ROS oxidizes Keap1, which is degraded by ubiquitin-dependent degradation and releases Nrf2. When Nrf2 is released, it travels to the nucleus to bind antioxidant response elements (ARE). Activation of the Nrf2/ARE pathway upregulates the transcription of several antioxidant enzymes, including HO-1, NADPH-dependent quinone reductase, and GSH synthesis enzymes like GR and glutamate-cysteine ligase modifier subunit (GCLM) [5].

Many antioxidants have been known to target MASLD, including vitamins E and C in MASLD patients, caffeine and coffee polyphenols in murine models of Western-style HFDs [14]. Other studies detail the use of metformin and Hesperetin in rat hepatocytes and HepG2 cells, and caffeine in zebrafish [14]. Mitochondria-targeting synthetic and naturally-occurring antioxidants like melatonin have an immense potential to treat or prevent MASLD [17].

#### Role of intestinal barrier dysfunction in MASLD

Earlier reports showed that intestinal barrier dysfunction plays a causal role in MASLD [160, 245–248]. In our opinion, intestinal barrier dysfunction in MASLD is caused by increased oxidative stress, which can cause apoptosis of gut epithelial cells (enterocytes), and PTMs of intestinal TJ/AJ proteins that lead to their decrements via ubiquitin-dependent proteolytic degradation [86]. WT mice exposed to a Western-style HFD (containing cholesterol to represent a fast food diet) showed elevated serum LPS within 2 weeks of feeding, indicating gut barrier dysfunction; insulin resistance, hepatic inflammation, and fibrosis followed at 10 and 22 weeks of feeding, suggesting a causal role of gut barrier dysfunction in the progression of liver disease [244]. [CYP2E1 levels may have been induced by endogenous ethanol production by gut microbiota [180, 249]. Although the cell death mechanisms of gut enterocytes in these rodent models of MASLD/MASH were not described, mitochondrial dysfunction may have happened due to elevated oxidative PTMs and Bax-mediated apoptosis. Thus, CYP2E1 may be an essential target to mitigate gut barrier dysfunction in MASLD/MASH [41, 232]. However, NADPH-oxidase may not be as important as CYP2E1 in the development of intestinal barrier dysfunction in MASH, as done in one study using a methionine and choline-deficient diet (MCD) [250].

### Toxicant-associated fatty liver disease

#### Carbon tetrachloride

Carbon tetrachloride (CCl_4_) has been widely used as a hepatotoxic agent in experimental models. Since 1924, scientists have understood that CCl_4_ causes acute hepatotoxicity, fatty liver, and liver fibrosis, depending on the dosage and treatment duration. In the last hundred years, scientists have understood this mechanism to include lipid peroxidation, hepatotoxicity, and liver damage through CYP2E1-mediated metabolism into toxic trichloromethyl and trichloromethyl peroxyl radicals; these toxic metabolites cause oxidative damage in the mitochondria and ER [78]. CCl_4_-mediated hepatotoxicity is exacerbated by a Western-style HFD [251] and alcohol consumption [252], which both happen to elevate CYP2E1 levels. Previous animal studies also found that *Cyp2e1*-KO mice were relatively resistant to CCl_4_-mediated hepatotoxicity [189, 253].

Despite many reports, the molecular mechanisms of CCl_4_-mediated hepatotoxicity and acute liver injury could be further elucidated. A recent report detailed the time-dependent events of PTMs and hepatotoxicity in WT versus *Cyp2e1*-KO mice [56]. The results showed that JNK-mediated phosphorylation of many mitochondrial proteins occurred 1–8 h in WT mice after CCl_4_ treatment [56]. At the same time, acute hepatotoxicity, assessed by serum ALT activity, LPO levels, and H&E-stained liver histology, was observed 24 hours after IP injection of a single toxic dose (50 mg/kg) of CCl_4_ [56]. In this model, activated p-JNK translocated to mitochondria at 2 h and phosphorylated many mitochondrial proteins, such as ALDH2, ubiquinone-dependent NADH dehydrogenase (Complex I), and α-ketoglutarate dehydrogenase, decreasing their activities [56]. These changes in protein phosphorylation, decreased activities, and liver injury were markedly prevented when CCl_4_-exposed WT mice were co-treated with a highly selective JNK inhibitor (i.e., SU3327 or BI-78D3) and mitochondria-targeted Mito-TEMPO [56]. This model also demonstrated that *Cyp2e1*-KO mice were protected from CCl_4_-mediated cellular changes, JNK-mediated phosphorylation, mitochondrial dysfunction, and liver injury [56]. Thus, CYP2E1-mediated metabolic activation of CCl_4_ was shown to play a significant role in ROS production, and JNK-mediated PTMs in promoting mitochondrial dysfunction and acute liver injury. In fact, increased oxidative stress stimulated the activation of JNK, which translocated to mitochondria and phosphorylated many target proteins ( decreasing their activities), leading to hepatotoxicity at a later time point. These results support the causal role of PTMs in promoting mitochondrial dysfunction and the characteristic hepatotoxicity of TAFLD.

#### Thioacetamide

Thioacetamide (TAA) was developed as an anti-fungal agent. However, TAA exposure has caused acute liver injury, cirrhosis, and HCC, in experimental models [254–257] and humans [257], depending on the dosage and TAA exposure duration [255, 258]. TAA-mediated hepatotoxicity and other tissue damage, including renal and cardiac toxicity, are believed to be induced through CYP2E1-mediated TAA metabolism in mammals. In fact, *Cyp2e1*-KO mice were protected from TAA-mediated hepatotoxicity and HCC [259]. A recent report showed that TAA-mediated hepatocyte pyroptosis in mice can be attenuated by administration of an anaerobic bacterial species named *Parabacteroides distasonis* by modulating intestinal bile acid metabolism [260]. In this report, decreased levels of *Parabacteroides distasonis* were observed in people with hepatic fibrosis. Administration of this bacteria inhibited bile salt hydrolase and suppressed intestinal expression of Farnesoid X receptor (FXR) and its signaling [261]. It also reduced hepatic levels of a component of bile acid named taurochenodeoxycholic acid (TCDCA), which typically induces mitochondrial permeability transitions and caspase-11-dependent pyroptosis; therefore, reduction of TCDCA mitigated TAA-mediated liver fibrosis in mice [261]. Additionally, co-administration of the natural compound celastrol increased the relative abundance of *Parabacteroides distasonis,* promoting bile acid excretion and hepatic fibrosis attenuation. Celastrol was also shown to increase SIRT1 expression and FXR signaling to improve cholestatic liver disease [261]. These results suggest the beneficial effects of *Parabacteroides distasonis* and celastrol against liver disease and suggest crosstalk between the gut microbiota and liver disease. Based on these results, more studies detailing gut–liver interactions are needed to improve liver disease prognosis.

### Drug-induced liver injuries

Drug-induced liver injuries (DILI) account for 50% of all acute liver diseases. Of this 50%, 37% of DILI are associated with acetaminophen (APAP, paracetamol) and the other 13% are caused by isoniazid (isonicotinic acid hydrazide, INAH), TAA, erythromycin, diclofenac, and others [262]. DILI cases fall into two major categories: intrinsic/direct hepatotoxicity or idiosyncratic hepatotoxicity, with a third emerging category being indirect mechanisms of hepatotoxicity (that may include gut dysbiosis) [263]. The most common cause of intrinsic DILI is APAP overdose mechanistically through mitochondrial dysfunction and hepatocyte damage [4], and the severity of idiosyncratic DILI varies based on the geographical region of prevalence [264] and environmental factors such as alcohol consumption and other pathological conditions, including obesity, insulin resistance, and (pre)diabetes. For example, the leading cause (45.4% according to the American DILI Network) of idiosyncratic DILI cases in the US and UK were due to antibiotic use, followed by herbal and dietary supplements, whereas, in Korea, herbal and dietary supplements were the cause of 70% of idiosyncratic DILI cases [142, 264]. The National Institutes of Health (NIH) supplies a database named LiverTox (http://livertox.nih.gov) that one meta-analysis [265] grouped into categories based on types of hepatotoxicity and should provide more detailed information on idiosyncratic cases. However, this review will focus on a few models illustrating the mechanisms of intrinsic cases of DILI.

#### Over-the-counter pain medicines: acetaminophen

APAP, the active ingredient found in Tylenol, Panamax, Excedrin, and Panadol, is generally prescribed and available as an over-the-counter medicine to treat pain, fever, and inflammation by reducing the production of prostaglandins. However, APAP overdose is the leading cause of acute liver diseases in the UK and USA [262, 266] and is responsible for 50% of acute DILI in the USA [267]. A multitude of investigations have elucidated the critical role of oxidative stress, mitochondrial dysfunction, and hepatocyte death in the mechanism of APAP overdose-induced liver diseases [110, 267–270]. In normal doses, APAP toxicity is not observed because its toxic metabolites are neutralized by GSH. However, after fasting (which decreases GSH), large amounts of APAP rapidly deplete cellular GSH, leading to acute liver injury [110, 268, 269]. In one mechanism, APAP becomes hepatotoxic after its metabolism by CYP2E1 and other P450 isoforms, and produces a reactive metabolite named *N*-acetyl-*p*-benzoquinone imine (NAPQI) [110, 271, 272]. This reactive metabolite produces conjugation adducts for many cellular proteins, including those involved in the mitochondrial ETC and others [110, 268, 269]. These protein-adducts, in turn, create mitochondrial nitro-oxidative stress, likely promoting PTMs of mitochondrial proteins [26, 41, 68, 178, 244], leading to impaired mitochondrial function and energy production. However, the roles of NAPQI-related covalent protein adducts have been challenged with evidence of similar patterns of protein-adducts observed in studies with a non-hepatotoxic structural analog named 3’-hydroxyacetanilide [26, 41, 68, 178, 244, 273]. In one study, pretreatment with gadolinium chloride, a suppressor of Kupffer cells, significantly prevented APAP-mediated liver injury but not NAPQI-protein adducts, suggesting a noncritical role of NAPQI-protein adducts in APAP-related hepatotoxicity [274]. Thus, scientists have explored the role of various PTMs in APAP-mediated acute hepatotoxicity. APAP can trigger a mitogen-activated protein kinase (MAPK) cascade [269], ultimately activating c-Jun N-terminal kinase (JNK) phosphorylation [267, 268, 271]. Phosphorylated JNK travels into the mitochondria to further phosphorylate many mitochondrial proteins, including the mitochondrial ETC, causing even more ROS leakage and oxidative stress by binding to SH3 homology associated BTK binding protein (Sab) [110, 275]. This excessive buildup of ROS causes a mitochondrial permeability change and the release of mitochondrial proteins that induce DNA damage and activate hepatocyte apoptosis [269, 276]. Ultimately, this process also impairs the autophagosome, leading to defective mitophagy [110] and receptor interacting protein (RIP) kinase-mediated necrosis of the cell [110, 268, 269, 277–279].

In addition to JNK-mediated protein phosphorylation, the essential roles of nitrated proteins in mitochondria and cytosol were reported [53, 59]. In these studies, time-dependent events of protein nitration and necrotic cell death were compared after a single toxic dose (350–400 mg/kg) of APAP was administered to WT and *Cyp2e1*-KO mice. Nitrated cytosolic and mitochondrial proteins were observed around 2–4 h, and hepatocyte necrosis and elevated serum ALT levels were noticed 24 hours after APAP exposure in WT mice. Mitochondrial ALDH2, ATP synthetase, GPx, 3-ketoacyl-CoA thiolase (KAT), SOD2, and cytosolic SOD1 were nitrated, and their activities were suppressed at 2–4 hours, suggesting a causal role of protein nitration in promoting mitochondrial dysfunction, leading to apoptosis or necrosis of hepatocytes. Additionally, the non-toxic analog 3’-hydroxyacetanilide did not cause nitration and liver injury in WT mice. In contrast, protein nitration and hepatotoxicity were not seen in the corresponding *Cyp2e1*-KO mice, supporting the vital roles of CYP2E1 and nitration in APAP-mediated DILI [53, 59]. Recent studies suggest that boosting antioxidants in the mitochondria through mitoquinone [280] and others [270] may serve as effective treatments for APAP-induced DILI.

It is also known that APAP toxicity is enhanced by co-existing conditions such as obesity and MASLD [281] and is often potentiated by alcohol intake [282–285]. APAP toxicity is also observed in people with alcohol use disorder (AUD), possibly due to upregulated CYP2E1 activity [282, 286, 287] or a response to fasting [288] (which is known to decrease GSH levels and increase CYP2E1 [202]). Thus, co-administration of excessive alcohol and therapeutic doses of APAP may put individuals at risk of severe liver injury [289].

#### Misused substances: cocaine, amphetamines, and MDMA

It has been well-established that many misused substances such as pain-relieving drugs, mood-enhancing drugs, and recreational psychostimulants such as cocaine, amphetamines, and 3,4-methylenedioxymethamphetamine (MDMA, Ecstasy, Molly) are known to cause significant toxicity to the liver as well as many other organs [290]. Cocaine toxicity is mediated by its oxidative metabolism by P450 isozymes, including CYP2B, CYP3a, and CYP2E1. Reactive metabolites of cocaine (i.e., norcocaine and norcocaine nitric oxide) and increased ROS produced during P450-mediated catalysis are known to increase oxidative stress, leading to mitochondrial dysfunction, hepatocyte death, and liver injury [77, 291]. Hepatotoxicity due to cocaine has been significantly worsened in the presence of other agents such as endotoxin [292] and alcohol; co-administration of cocaine and ethanol produces potently toxic cocaethylene [293] and suppresses mitochondrial ALDH2 activity through PTMs [294]. Although the detailed cell death signaling mechanisms are unknown, one recent report showed that cocaine toxicity was attenuated in p53-knockout (p53-KO) mice, suggesting the involvement of the p53-mediated apoptosis pathway [295]. A recent study showed that cocaine caused mitochondrial dysfunction and acute liver injury in WT mice, and hepatotoxicity was prominently observed in *Gpx-1*-KO mice but protected in Gpx1-overexpressing transgenic mice [66]. Based on the importance of PTMs in mitochondrial dysfunction and hepatocyte cell death in DILI, ALD, and MASLD models, the contributing roles of various PTMs in cocaine-mediated mitochondrial dysfunction and hepatotoxicity are expected, although this needs to be verified by future research.

Overdoses of amphetamine-type psychostimulants like amphetamines and MDMA can cause hyperthermia, tissue injury, acute liver failure, and death, depending on their dosage and host conditions (e.g., hepatic GSH levels). MDMA toxicity is thought to occur through the P450-mediated production of its reactive metabolites, which can activate lysosomal functionand increase mitochondrial swelling and dysfunction [103, 296]. Other risk factors, such as hyperthermia,elevated neurotransmitter effluxes, increased LPOs, oxidized biogenic amines, decreased GSH, and dysregulated host environments, have also been suggested [103]. Although the detailed mechanisms of tissue injury are poorly understood, it is widely accepted that increased oxidative stress and nitrative stress play a key role in promoting MDMA-mediated hepatotoxicity. Targeted proteomics approaches revealed that many mitochondrial and cytosolic proteins were oxidatively modified upon exposure to MDMA, and some of them, like mitochondrial ALDH2, 3-ketoacyl-CoA, and ATP synthetase, were inactivated [198]. These reports support the role of oxidatively modified cellular proteins in promoting mitochondrial dysfunction and ER stress, contributing to cell death of hepatocytes and liver injury [49, 296, 297]. Other types of PTMs, such as nitration, JNK-mediated phosphorylation, and acetylation, of proteins, can also take part in the pathology of MDMA-mediated tissue injury, although this area solicits further investigation.

#### Anti-cancer agents: cisplatin

Many chemotherapies have been found to cause hepatotoxicity as well as other tissues. Antitumor antibiotics (e.g., dactinomycin, doxorubicin, and mitomycin) and alkylating agents (e.g., Busulfan, Melphalan, and Cyclophosphamide) have been shown to elevate serum ALT and AST activities in liver function tests [298]. Platinum-based agents (e.g., Oxaliplatin, Cisplatin, and Carboplatin) have also been shown to elevate serum levels of liver transaminases to cause steatohepatitis [298]. Cisplatin is a platinum-based chemotherapy drug that was approved by the FDA in 1978 despite its harsh side effects of inducing organ damage (including the liver and kidneys) [299], through oxidative metabolism via CYP2E1 and CYP4A11 [300]. Since then, the effects of cisplatin have been studied and reviewed; cisplatin induces oxidative stress [301–303], inflammation [299, 301, 304], mitochondrial dysfunction [304], apoptosis [305], and DNA damage [299, 304, 306]. Cisplatin causes oxidative stress by increasing MDA and decreasing GSH, GPx, CAT, and SOD [307]. Cisplatin also stimulates apoptotic signaling pathways involving TNF-α, Bax and Bcl-2, cytochrome c, and caspase-3, and stimulates IL-6 related inflammatory pathways [307].

There are also theories on how cisplatin-induced hepatotoxicity happens through the gut–liver axis. One study mentioned that cisplatin-induced liver toxicity is accelerated by inflammation and oxidative stress in the gut through the increased abundance of pathological bacteria like *Escherichia, Parabacteroides,* and *Ruminococcus*, all of which are Gram-negative bacteria [301]. In this study, antibiotic treatment improved liver histology, promoted Nrf2 activation, increased the levels of GSH, and inhibited the JNK- and p38-related cell death signaling pathways [301, 308], demonstrating its significance as an effective treatment for cisplatin-mediated hepatotoxicity.

#### Anti-tuberculosis agent: isoniazid

It is known that an anti-tuberculosis agent, isoniazid (isonicotinic acid hydrazide, INAH), can cause hepatitis through CYP2E1-mediated metabolism [309]**.** INAH or INAH metabolites, such as hydrazine, can cause mitochondrial injury, mitochondrial oxidative stress, and impaired metabolic homeostasis [310]. Consequently, these reactive metabolites and ROS will likely promote oxidative PTMs, lipid peroxidation, and cell death pathways. In addition, these reactive metabolites can bind to proteins, lipids, or nucleic acids and inhibit the enzymes in the mitochondrial ETC, resulting in oxidative stress, mitochondrial dysfunction, and hepatotoxicity [310, 311].

Like cisplatin, INAH alters CAT activity, GPx activity, and GSH content while increasing ROS and MDA levels. It also decreases the expression of microRNA-122 and PPAR-α and increases AP1 and JNK phosphorylation [312]. It also upregulates the mRNA expression of ER stress-related factors, including glucose-related protein 78 (Grp78), activating-transcription-factor-6 (ATF6), protein kinase RNA-like ER kinase (PERK), inositol-requiring enzyme 1 (IRE1), x-box binding protein 1 (XBP1s), and C/EBP homologous protein (CHOP) [312]. Furthermore, it upregulates apoptotic signaling pathways, including Bax, cytochrome c release from mitochondria, and activation of caspases 3, 8, and 9. Lastly, it suppresses the Nrf2 signaling pathway, including Nrf2 and its downstream targets of heme oxygenase-1 (HO1), NAD(P)H quinone dehydrogenase 1 (NQO1), GCLM, and glutamate-cysteine ligase catalytic subunit (GCLC) [312].

#### Anti-depressants and anti-psychotics

Recent reports have shown that many anti-depressant medications clinically used are known to cause side effects of liver toxicity and weight gain [313, 314]. The mechanisms of these undesirable effects are poorly understood. Most of these side effects are idiosyncratic hepatotoxicity, and their symptoms appear as early as 5 days and last up to 3 years. Some severe cases are linked to users’ deaths [313], possibly due to severe drug interactions with other agents or pre-existing conditions like metabolic syndrome, obesity, and diabetes [315]. In contrast, people with MASLD may be prone to developing anxiety and depression [316]. Thus, this newly emerging area with anti-depressants related liver injury needs more studies.

Much of depression, schizophrenia, bipolar disorders, and psychotic disorders are associated with oxidative stress in the brain through the gut–brain axis [317, 318]. However, it is also known that the metabolisms of anti-depressants, including monoamine oxidase inhibitors (MAOIs), tricyclic antidepressants, selective serotonin reuptake inhibitors (SSRIs), first-generation anti-psychotics (FGAs), and second-generation anti-psychotics (SGAs), cause oxidative stress which may affect mitochondrial functions [314]. The mechanism by which anti-depressant and anti-psychotic medications induce oxidative stress in hepatocytes begins with their metabolism through cytochrome P450 isoforms, causing reduced GSH to convert to oxidized glutathione disulfide (GSSG), as well as producing ROS and reactive metabolites that covalently bind proteins, lipids, and nucleic acids [314]. These changes affect multiple mitochondrial metabolism pathways, including the proton gradient in oxidative phosphorylation and the proportion of  superoxide anion metabolites leaking from the ETC [314]. These ROS activate apoptotic pathways involving Bax and Bcl-2, cytochrome c release from mitochondria, and activation of caspase-3 cleavage, as well as inducing cell damage and necrosis [314]. They can also decrease the activities of GST, GPx, and CAT [314]. A recent report also showed that a tricyclic anti-depressant named clomipramine caused mitochondrial dysfunction by decreasing ATP production [319]. It is important to note that all anti-depressants and anti-psychotics collectively follow this umbrella mechanism, although there are some subtle differences in individual mechanisms of creating hepatotoxic oxidative stress [314]. Oxidative stress is also produced from inflammatory pathways induced by chronic usage of anti-depressants and anti-psychotics; DAMP signals are likely to stimulate the apoptotic and necrotic pathways [314]. They also activate Kupffer cells through TLRs and inflammatory signaling pathway, including NF-κB, tumor necrosis factor-α (TNF-α), ROS, nitric oxide (NO), and chemokine and cytokine storms [314]. Chemokine and cytokine storms can also recruit circulating lymphocytes, eosinophils, and neutrophils, infiltrating the liver to start further inflammation and hepatotoxicity [314].

### Viral hepatitis

It is also known that infection with hepatitis B (HBV) and C virus (HCV) can cause mitochondrial dysfunction, hepatic inflammation, and chronic liver disease, in people and cell lines, through the hepatitis virus core and other proteins associated with HBV/HCV [16, 104]. For instance, over-expression of the HCV core protein has been shown to induce oxidative stress and mitochondrial depolarization, leading to cell death [104]. These events were Ca^2+^-dependent and could be prevented by Ca^2+^ chelation. In addition, chronic infection with HCV increased oxidative DNA damage with high levels of MDA and 4-HNE, which correlated with the degree of liver inflammation and fibrosis [320]. The activity of the mitochondrial complex I enzyme (NADH-ubiquinone oxidoreductase) was suppressed in the genomic HCV replicon cells. This suppression decreased mitochondrial GSH and increased ROS production; these changes were restored by decreasing HCV replication with Fluvastatin [321]. Another study showed that the HCV core protein and CYP2E1, which produces ROS, work additively to decrease mitochondrial GSH and sensitize hepatocytes to ROS-mediated cell death [322]. Furthermore, a retrospective study with HCV-infected patients before and after antiviral treatment revealed that higher levels of serum LPS and intestinal fatty acid binding proteins, markers of intestinal permeability, were observed in patients with fibrosis/cirrhosis than those of patients without fibrosis and healthy volunteers [149] in both HBV [162–165] and HCV infections [166–168]. These studies indicate the importance of intestinal barrier dysfunction in accelerating hepatitis virus-induced liver disease outcomes through the gut–liver axis.

### Hepatocellular carcinoma (HCC)

ALD and MASLD have been known to ultimately progress to HCC; 4.4 per 100,000 people with MASLD were diagnosed with HCC globally from 1989 to 2015 [221]. HCC is highly associated with metabolic risk factors and alcohol consumption [323], and is exacerbated by underlying cirrhosis [324]. Humans with the ALDH2*2 gene variants are more susceptible to alcohol-induced HCC [325] and esophageal cancers [73, 326]. ALDH2*2 protein variants are also individually-correlated with HCC [327, 328] and are identified as potential biomarkers for HCC [329, 330]. Some believe that accumulated acetaldehyde, LPOs, and ROS are carcinogenic and contribute to HCC through aging; this may allow fibrosis to progress to HCC by modulating various PTMs that increase DNA adducts of liver cells [9, 331]. Alcohol-mediated DNA oxidation, resulting from the polymorphisms of the *ALDH2* gene and activation of CYP2E1, may also partake in the development of HCC and other cancers [9, 194, 331–333]. Overall, excessive alcohol intake induces CYP2E1 and/or inactivates mitochondrial ALDH2; both can lead to oxidative DNA damage, apoptosis, suppressed cell proliferation, and altered inflammatory pathways [9, 331], contributing to the development of tumors. Thus, targeting CYP2E1 or activating ALDH2 with safe chemicals, including naturally occurring dietary supplements [334], is a promising strategy to prevent alcohol-induced HCC. Recent reports showed that CMZ, a specific inhibitor of CYP2E1, was shown to prevent or improve ALD in humans [335] and experimental models of fibrosis and HCC [192]. Oxidative stress and dysbiosis are critical factors contributing to the progression of ALD to HCC [169, 331, 336, 337], and thus serve as important targets to prevent the onset of alcohol-related HCC [336, 338].

## Challenges and opportunities

### Challenges

One challenge is that many overlapping similarities exist between ALD and other liver diseases [339], but treatments for each liver disease can differ. A practical guideline for treating ALD patients described the clinical observation that ALD frequently occurred with other liver diseases, including MASLD and HCV [340]. Specific studies have been designed to distinguish the characteristics of ALD and MASLD. However, their histological features and pathological mechanisms are very similar [341]; this is because many metabolic risk factors for MASLD/MASH overlap with those of ALD [342] (and significantly increase the risk of severe liver disease [343]) and patients need to be treated for both types of liver disease.

Another challenge is the additive or synergistic interactions between alcohol intake and many other risk factors for liver diseases. For instance, Åberg et al. showed that mild and moderate consumers of alcohol with obesity, waist circumference, and diabetes may have increased levels of liver disease; in contrast, heavy drinkers with diabetes, large waist circumferences, and high BMIs had elevated risks of severe liver disease [343]. Other studies have detailed that BMI and moderate alcohol intake increase the risk of liver disease [344, 345]. Raynard et al. showed that BMI and blood glucose are independent risk factors for alcohol-associated fibrosis (cirrhosis) [342]. However, more studies are needed to differentiate the risk factors for ALD and MASLD. Due to the synergistic or potentiation effects of concurrent exposures to alcohol, HFDs, smoking, recreational and pharmaceutical drugs, individual mechanisms of liver injury and disease listed in previous sections may not be observed in isolation.

Likewise, a third consideration is that each of these exposures promotes unique patterns of ROS/RNS, inflammation, dysbiosis, and PTMs, and most of these features take place simultaneously. Overlapping pathological risk factors of liver diseases could further increase the difficulty of prognosis and therapeutic benefits unless those multiple interventions target many pathways simultaneously.

A fourth consideration is that the pharmacokinetics, specifically the absorption, distribution, metabolism, excretion, and toxicity (ADME-Tox) properties of hepatotoxic compounds and targeted interventions vary. These complexities present a significant challenge in the context of DILI. However, the Liver Tox Knowledge Base (LTKB) may provide more insight into adverse drug reactions in DILI research [255] and allow medical professionals to distinguish DILI caused by agents with different latencies, symptoms, mechanisms, and responses [346]. An editorial suggests that digitalizing these assessments differentiating DILI from ALD and MASLD  and detecting specific agents of hepatotoxicity with a Revise Electronic Causality Assessment Method (RECAM) may improve the likelihood of treating emergency cases of DILI [346, 347]. Others mention the possibility of artificial intelligence (AI) for DILI predictions [348], but additional research is needed in the emerging field of AI.

The final challenge in liver disease research is establishing the correct diagnosis to differentiate between various liver diseases. Serum enzymes that are elevated in ALD may not necessarily be elevated in DILI diagnoses. Additionally, the current diagnosis of many liver diseases, such as MASLD and MASH, involves invasive access to biomarkers, often requiring biopsy surgeries for targeted interventions [349]. Some studies suggest screening for MASLD using less-invasive procedures (i.e., ultrasounds) [350], but deciding on a screening method may prove complex given the varied cost of diagnosis and treatment under different health insurance plans.

### Opportunities

Fortunately, many preventions and treatments are being investigated for various liver diseases. Other than what has already been mentioned, interventions for MASLD include but are not limited to four main specific targets. The first intervention class targets hepatic fat accumulation by modulating PPARs (i.e., pemafibrate and elafibranor), targeting FXR signaling (i.e., obeticholic acid and celastrol), inhibiting *de novo* lipogenesis (i.e., aramchol and ACC inhibitors), and utilizing fibroblast growth factor analogues [347]. The second intervention class aims to alleviate oxidative stress, inflammation, and apoptosis, including ASK1 and caspase inhibitors (i.e., Emricasan) [347]. The third intervention class targets the intestinal microbiome and metabolic endotoxemia through IMMe124, TLR antagonists, and antibiotics (i.e., solithromycin). The fourth intervention class works to mitigate hepatic fibrosis through antifibrotic agents [i.e., cenicriviroc, (a cysteine-cysteine-motif chemokine receptor-2,5 antagonist) and gelectin-3 antagonists] [347].

Current interventions for ALD include abstinence, nutritional support, glucocorticosteroids, Pentoxifylline, anti-TNF therapy, antioxidants, liver transplantations, probiotics, antibiotics, *S*-adenosyl methionine, betaine, endocannabinoid antagonists, osteopontin inhibitors, and stem cell therapy, as reviewed [351]. To facilitate the development of safe and effective therapeutic drugs for treating ALD patients, the National Institute on Alcoholism and Alcohol Abuse (NIAAA, NIH) supports a multi-center Consortia for drug evaluation studies in double-blind randomized clinical trials. Since the start of the multi-center consortia in the early 2010s, many reports on clinical trials have been published. In addition, the executive summary of many published reports and other information related to various clinical trials by the multi-center Consortia, including DASH (Defeat Alcoholic Steatohepatitis) and TREAT (Translational Research and Evolving Alcoholic Hepatitis Treatment), have been compiled and available in Alcoholic Hepatitis Network (AlcHepNet). Currently, three agents, anakinra as an inhibitor of the IL-1 receptor [352], prednisone [353, 354], zinc sulfate [355], and coffee [356] are being evaluated for their efficacies in phase II clinical studies for alcohol-associated hepatitis. We expect more reports on the clinical trial outcomes to be published by the Consortia in the future. However, the most crucial factor for long-term survival and improvement of ALD patients is abstinence without relapse of AUD. Thus, many scientists proposed that ALD patients be treated with integrated care by preventing AUD [357] and obtaining nutritional support [358–361].

MASLD and ALD prevention may also begin with identifying bacterial signatures that distinguish the two and differentially diagnosing each through fecal samples [362]. Many other natural compounds such as silymarin, resveratrol, curcumin, and berberine have effectively prevented the progression of MASLD and MASH [363, 364]. Some of these naturally occurring compounds function as *antioxidants* and inhibitors of CYP2E1 [365–367] to prevent ALD, MASLD, and DILI. In addition, many naturally occurring antioxidants, including resveratrol, quercetin, and curcumin, are known to activate sirtuins, Nrf2, and PGC1α to improve liver disease outcomes [85, 138, 139, 363, 364, 368]. Based on the previous information regarding the prevalence of sirtuins 3, 4, and 5 in mitochondria and protectors against liver diseases and general age-related diseases, sirtuin activators including naturally occurring polyphenols would exhibit great therapeutic benefits [86, 126, 369]. Additionally, given the plethora of studies suggesting that SIRT1 is a therapeutic target in ALD, SIRT1 is another opportunity for precision medicine and targeted interventions [370]. Lastly, ghrelin has been shown to improve oxidative stress, apoptosis, and inflammation in MASLD development [371].

DILI may be prevented with adequate nutrition and abstinence. It may also be prevented by predicting its onset and type; this may be done by measuring hepatic transporter inhibition, mitochondrial toxicity, reactive metabolite formation, hepatocyte cytotoxicity, as well as the dose and physiochemical properties of the drug ingested [372]. Current interventions for DILI include abstinence, *N*-acetylcysteine, corticosteroids, ursodeoxycholic acid, silymarin, glycyrrhizin, bile acid washouts, and emergency liver transplants [373].

#### Modulation of mitochondrial dysfunction, PTMs, and oxidative stress for liver diseases

Overall, mitochondrial dysfunction is associated with decreased function of energy supply, redox homeostasis, fat oxidation, cellular metabolism, and cell survival signals [374]. Webb et al. detailed that targeting mitochondrial dysfunction to prevent liver disease progressions includes altering metabolic signaling cascades to increase energetic efficiency, mitigating ROS/RNS, and restoring mitophagy and other systems to maintain homeostasis within mitochondria [91]. There are many ways to treat mitochondrial dysfunction liver diseases, including the consumption of a less caloric diet, anti-diabetic drugs (i.e., elafibranor, liraglutide, metformin, thiazolidinediones, MSDC 0602K), bile acid regulators (i.e., obeticholic acid, ursodeoxycholic acid), and antioxidants that act on nuclear receptors or mitochondrial metabolism (i.e., vitamin E, tempol, resveratrol) [91, 375]. Other mitochondria-targeted antioxidants, including mitoquinone [280], MitoE [376], mitoquinol mesylate (Mito-MES) [377], mitochondria-targeted ubiquinone [52], and quercetin [378] were reported to affect mitophagy and improve liver conditions. Furthermore, silymarin, corilagin, anthocyanins, dihydromyricetin, berberine, hydroxytyrosol, cysteamine, pentoxifylline, avocado oil, and pegbelfermin, mitotherapy, as well as ACC inhibitors, genistein, and aramchol were reported to improve mitochondrial function in MASLD [237]. Naturally occurring terpenoid polyphenols, including capsaicin [140], are excellent liver disease preventions that target mitochondrial dysfunction [379, 380] and CYP2E1-induced oxidative stress [381]. *N*-acetylcysteine [141], naturally occurring terpenoid polyphenols, and phenolic acids [368] were reported to minimize oxidative stress and hepatotoxicity by targeting antioxidant enzymes [299]. Melatonin is another promising agent that improves mitochondrial abnormalities in APAP-induced DILI [382, 383], chemotherapies [384], and other liver injuries [385].

#### Targeting intestinal barrier dysfunction for liver diseases

Gut dysbiosis is responsible for the exacerbation and progression of various liver diseases [386] through the promotion of toxic metabolites [150]; in fact, targeting gut dysbiosis has the potential to mitigate the onset and progression of liver diseases [150, 387]. Given this information, it is necessary to create intestinal eubiosis or symbiosis to mitigate liver disease development and progression. Many gut bacteria are helpful in their ability to turn toxic compounds into non-toxic compounds; for instance, *B. xylanisolvens* can metabolize nicotine in smokers with MASLD, preventing the progression to MASH [387].

Many reports reviewed different mechanisms by which the gut can be targeted to prevent liver disease or metabolic diseases caused by intestinal barrier disruption and endotoxemia. Considering the critical connection of the gut microbiota and liver, fecal microbiota transplants (FMT), or administering probiotics of *Lactobacillus rhamnosus* GG [388] and *Akkermansia muciniphila* [389] may be used to treat ALD [390] or metabolic disease in general [391]. In addition to probiotics, other gut-targeted treatments include antibiotics, prebiotics, and “postbiotics”. These treatments have various targets, acting on the microbial barrier, physical barrier, or chemical barrier of the intestines [152, 392]. Modulation of the microbial composition of the intestines would decrease the production and release of LPS into serum [393], regulate FXR signaling [394], limit or lower bacterial metabolites such as trimethylamine (TMA) and trimethylamine-*N-*oxide [395, 396], and prevent oxidative stress, inflammation, and gut barrier dysfunction [393] (Table 1).

**Table 1:** Opportunities to treat liver diseases through various targets

Designed to target	ALD	MASLD	DILI
Mitochondrial dysfunction	Abstinence [351] Nutritional support: Vitamins like folate, vitamin B6, vitamin B12, vitamin A, and thiamine [351] Minerals (like selenium, zinc, copper, and magnesium [351] Nicotinamide riboside [85] Mitochondria-targeted ubiquinone [52]	Silymarin, corilagin, anthocyanins, dihydromyricetin, berberine, hydroxytyrosol, cysteamine, pentoxifylline, avocado oil, and pegbelfermin, mitotherapy, as well as ACC inhibitors, genistein, and aramchol [237] Ursodeoxycholic acid [373] Less caloric diet, anti-diabetic drugs (i.e., elafibranor, liraglutide, metformin, thiazolidinediones, and MSDC 0602 K) [91, 375] MitoE [376] Mitoquinol mesylate (Mito-MES) [377] Capsaicin for septic acute liver injury [140]	Adequate nutrition and abstinence [372] Predicting its onset and type by measuring hepatic transporter inhibition, mitochondrial toxicity, reactive metabolite formation, hepatocyte cytotoxicity, as well as the dose and physiochemical properties of the drug ingested [372] Melatonin for chemotherapy hepatotoxicity [382] [384] Mitoquinone for acetaminophen hepatotoxicity [280]
Oxidative stress	Abstinence [351] Nutritional support: Vitamins like folate, vitamin B6, vitamin B12, vitamin A, thiamine [351] Minerals (like selenium, zinc, copper, and magnesium [351] Antioxidants [351] Nicotinamide riboside (targets SIRT1)[139] Melatonin and *N*-acetylcysteine [385] Mitochondria-targeted ubiquinone [52]	ASK1 and caspase inhibitors (i.e., emricasan) [347] Silymarin, resveratrol, curcumin, and berberine [363, 364] Ghrelin [371] Berberine [363] Melatonin and *N*-acetylcysteine [385] Quercetin [368, 378] Various flavonoids for CYP2E1-induced oxidative stress [381]	Adequate nutrition and abstinence [372] Predicting its onset and type by measuring hepatic transporter inhibition, mitochondrial toxicity, reactive metabolite formation, hepatocyte cytotoxicity, as well as the dose and physiochemical properties of the drug ingested [372] *N*-acetylcysteine [141, 373, 397] Silymarin [365–367, 373] Melatonin for chemotherapy hepatotoxicity [384] Melatonin with *N*-acetylcysteine for many types of DILI [385] Plant extracts and oil rich in flavonoids, terpenoids, polyphenols, and phenolic acids for cisplatin-mediated hepatoxicity [299]
PTMs	Abstinence [351] Nutritional support: Vitamins like folate, vitamin B6, vitamin B12, vitamin A, thiamine [351] Minerals (like selenium, zinc, copper, and magnesium [351] Naturally occurring polyphenols to target SIRT3, SIRT4, and SIRT5 [11, 58, 86, 126, 369] SIRT1 targets [370]		Adequate nutrition and abstinence [372] Predicting its onset and type by measuring hepatic transporter inhibition, mitochondrial toxicity, reactive metabolite formation, hepatocyte cytotoxicity, as well as the dose and physiochemical properties of the drug ingested [372]
Apoptosis	Abstinence [351] Nutritional support: vitamins like folate, vitamin B6, vitamin B12, vitamin A, thiamine [351] Minerals like selenium, zinc, copper, and magnesium [351]	ASK1 and caspase inhibitors (i.e., Emricasan) [347] Ghrelin [371] Quercetin [378] Capsaicin for septic acute liver injury [140]	Adequate nutrition and abstinence [372] Predicting its onset and type by measuring hepatic transporter inhibition, mitochondrial toxicity, reactive metabolite formation, hepatocyte cytotoxicity, as well as the dose and physiochemical properties of the drug ingested [372] Melatonin for acetaminophen hepatotoxicity [382, 383]
Dysbiosis/intestinal barrier dysfunction/endotoxemia	Abstinence [351] Nutritional support: vitamins like folate, vitamin B6, vitamin B12, vitamin A, thiamine [351] Minerals (like selenium, zinc, copper, and magnesium [351] Probiotics and antibiotics [351] Fecal microbiota transplants (FMT), or administering probiotics of *Lactobacillus rhamnosus* GG [388] and *Akkermansia muciniphila* [389, 390] Nicotinamide riboside [138]	IMMe124 [347] TLR antagonists [347] Antibiotics (i.e., solithromycin) [347] Fecal samples to identify bacterial signature differences from ALD [362] *B. xylanisolvens* can metabolize nicotine in smokers with MASLD, preventing progression into MASH [387] Resveratrol [363] Silymarin, curcumin, and berberine [363, 364]	Adequate nutrition and abstinence [372] Predicting its onset and type by measuring hepatic transporter inhibition, mitochondrial toxicity, reactive metabolite formation, hepatocyte cytotoxicity, as well as the dose and physiochemical properties of the drug ingested [372]
Inflammation	Abstinence [351] Nutritional support: vitamins like folate, vitamin B6, vitamin B12, vitamin A, thiamine [351] Minerals like selenium, zinc, copper, and magnesium [351] Glucocorticosteroids [351] Pentoxifylline [351] Anti-TNF therapy [351] Betaine [351] Osteopontin inhibition [351] Stem cell therapy [351] Nicotinamide riboside (targets SIRT1) [139]	ASK1 and caspase inhibitors (i.e., emricasan) [347] Ghrelin [371] Silymarin [363] Resveratrol, curcumin, and berberine [363, 364] Quercetin [378]	Adequate nutrition and abstinence [372] Predicting its onset and type by measuring hepatic transporter inhibition, mitochondrial toxicity, reactive metabolite formation, hepatocyte cytotoxicity, as well as the dose and physiochemical properties of the drug ingested [372] Corticosteroids [373] Silymarin [373] Glycyrrhizin [373]
Fat accumulation	Abstinence [351] Nutritional support: vitamins like folate, vitamin B6, vitamin B12, vitamin A, thiamine [351] Minerals like selenium, zinc, copper, and magnesium [351] Betaine [351] Mitochondria-targeted ubiquinone [52]	Modulating PPARs (i.e., pemafibrate and elafibranor) [347] Targeting FXR signaling (i.e., obeticholic acid and celastrol) [347] Inhibiting de novo lipogenesis (i.e., aramchol and ACC inhibitors) [347] Utilizing fibroblast growth factor analogues [347] Bile acid regulators (i.e., obeticholic acid, ursodeoxycholic acid) [373]	Adequate nutrition and abstinence [372] Predicting its onset and type by measuring hepatic transporter inhibition, mitochondrial toxicity, reactive metabolite formation, hepatocyte cytotoxicity, as well as the dose and physiochemical properties of the drug ingested [372] Bile acid washouts [373]
Fibrosis	Abstinence [351] Nutritional support: vitamins like folate, vitamin B6, vitamin B12, vitamin A, thiamine [351] Minerals like selenium, zinc, copper, and magnesium [351] Pentoxifylline [351] Liver transplantation [351] Betaine [351] Stem cell therapy [351]	Antifibrotic agents (i.e., cenicriviroc, (a cysteine-cysteine-motif chemokine receptor-2,5 antagonist)) [347] Gelectin-3 antagonists] [347] Less caloric diet, anti-diabetic drugs (i.e., elafibranor, liraglutide, metformin, thiazolidinediones, MSDC 0602 K) [91, 375]	Adequate nutrition and abstinence [372] Predicting its onset and type by measuring hepatic transporter inhibition, mitochondrial toxicity, reactive metabolite formation, hepatocyte cytotoxicity, as well as the dose and physiochemical properties of the drug ingested [372] Silymarin [373] Inhibitors of CYP2E1 [365–367]
Hepatitis	Anakinra (IL-1 inhibitor) [352], prednisone [353, 354], zinc sulfate [355], and coffee [356] are being evaluated for their efficacies in phase II clinical studies [352–356]		Adequate nutrition and abstinence [372] Predicting its onset and type by measuring hepatic transporter inhibition, mitochondrial toxicity, reactive metabolite formation, hepatocyte cytotoxicity, as well as the dose and physiochemical properties of the drug ingested [372]
Liver failure			Emergency liver transplants [373]

## Concluding remarks

This article briefly reviewed the causes and manifestations of various liver diseases, including ALD, MASLD/MASH, TAFLD, and DILI. We have precisely described the roles of oxidative stress and examples of PTMs (e.g., oxidation, *S*-nitrosylation, JNK-mediated phosphorylation, nitration, acetylation, and adduct formation) of mitochondrial proteins and transcription factors in promoting fat synthesis, mitochondrial dysfunction and apoptosis of hepatocytes, leading to individual liver diseases caused by different etiological agents. We have also explained the causal roles of CYP2E1 and NOXs as initial sources of ROS through the metabolism of many substrates, including alcohol (ethanol), long-chain FFAs, APAP, INAH, cisplatin, CCl_4_, cocaine, MDMA, antidepressants, and TAA. In addition, we have mentioned the protective role of mitochondrial ALDH2 against oxidative stress-mediated mitochondrial dysfunction and hepatotoxicity, apoptosis/necrosis of hepatocytes and gut enterocytes, the production of DAMPs, the activation of Kupffer cells and HSCs, altered inflammation, and fibrosis, leading to cirrhosis and HCC. We have also discussed the role of gut dysbiosis in promoting intestinal barrier dysfunction, endotoxemia, and liver disease through the gut–liver axis. Furthermore, we proposed four challenges regarding the diagnosis and prognosis of several liver diseases. Based on the mechanistic insights and challenges, we have suggested basic and translational research opportunities against liver diseases by listing the benefits of many agents, including naturally occurring antioxidants and synthetic compounds. We hope our review can contribute to developing new and effective preventive or therapeutic agents against individual liver diseases as well as organ damage in other tissues.

## Data Availability

Not applicable.

## Abbreviations

ADH: Alcohol dehydrogenase
AGE-adduct: Advanced glycation end product adduct
ALD: Alcohol-associated liver disease
AJ: Adherens junction
AMPK: AMP-activated protein kinase
ALDH2: Mitochondrial aldehyde dehydrogenase 2
APAP: Acetaminophen
ARE: Antioxidant response element
AUD: Alcohol use disorder
CAT: Catalase
CYP2E1: Ethanol-inducible cytochrome P450-2E1
DAMP: Danger-associated molecular pattern
DILI: Drug-induced liver injury
ER: Endoplasmic reticulum
ETC: Electron transport chain
FFA: Free fatty acid
FLD: Fatty liver disease
GPx: Glutathione peroxidase
GR: Glutathione reductase
GSH: Reduced glutathione
GSSG: Oxidized glutathione
HCC: Hepatocellular carcinoma
HBV: Hepatitis B virus
HCV: Hepatitis C virus
HFD: Western-style high-fat diet
4-HNE: 4-hydroxynonenal
HSC: Hepatic stellate cells
INAH: Isoniazid
JNK: C-Jun N-terminal protein kinase
Keap1: Kelch-like ECH-associated protein
KO: Knockout
LPO: Lipid peroxidation product
LPS: Lipopolysaccharide
MAPK: Mitogen-activated protein kinase
MASH: Metabolic dysfunction-associated steatohepatitis
MASLD: Metabolic dysfunction-associated steatotic liver disease
MDA: Malondialdehyde
MDMA: 3,4-methylenedioxy methamphetamine
MEOS: Microsomal ethanol-oxidizing system
NF-κB: Nuclear factor-κB
NO: Nitric oxide
NOX: NADPH-oxidase
Nrf2: Nuclear factor erythroid 2-related factor
PARP: Poly ADP-ribose polymerase
PGC-1α: Peroxisomal proliferator-activated receptor-γ coactivator-1α
PPAR-α: Peroxisomal proliferator-activated receptor-α
PTMs: Post-translational modifications
RNS: Reactive nitrogen species
ROS: Reactive oxygen species
α-SMA: α-Smooth muscle actin
SOD: Superoxide dismutase
SREBP-1: Sterol regulatory element-binding protein-1
TAA: Thioacetamide
TAFLD: Toxicant-associated fatty liver disease
TG: Triglyceride
TGF-β: Transforming growth factor-β
TJ: Tight junction
TLR4: Toll-like receptor 4
TNF-α: Tumor necrosis factor-α
SIRT1: NAD^+^-dependent deacetylase Sirtuin 1
VIM: Vimentin
